# Vaccination Is a Suitable Tool in the Control of Aujeszky’s Disease Outbreaks in Pigs Using a Population Dynamics P Systems Model

**DOI:** 10.3390/ani10050909

**Published:** 2020-05-24

**Authors:** Maria Angels Colomer, Antoni Margalida, Lorenzo Fraile

**Affiliations:** 1 Department of Mathematics ETSEA, University of Lleida, 25198 Lleida, Spain; colomer@matematica.udl.cat; 2 Institute for Game and Wildlife Research, IREC. Consejo Superior de Investigaciones Científicas-Universidad de Castilla la Mancha-Junta de Comunidad de Castilla la Mancha (CSIC-UCLM-JCCM), 13005 Ciudad Real, Spain; a.margalida@csic.es; 3 Department of Animal Science, ETSEA, University of Lleida, 25198 Lleida, Spain; 4 Agrotecnio, University of Lleida, 25198 Lleida, Spain

**Keywords:** pseudorabies, vaccination, management, control measures, Population Dynamics P system model

## Abstract

**Simple Summary:**

Maximizing the efficiency of pork production in line with sustainability and environmental restrictions presents a challenge for the pig industry in the coming years. It is necessary to develop practices based on cost/benefit analyses of the effects of disease on animal performance. Diseases can be controlled in various ways, such as vaccination programs and management protocols, among others, to control pathogens. We have developed a model to disentangle the effects of management and vaccination strategies to control one of the most important pig viral diseases, Aujeszky disease. Our results suggest that after confirming the diagnosis, early vaccination of most of the population is critical to decrease the spread of the virus and minimize its impact on pig productivity. However, the effect of management is negligible for the control of this virus. Thus, this model can be used to evaluate preventive medicine programs in the control of known diseases and for new ones that could appear in the future.

**Abstract:**

Aujeszky’s disease is one of the main pig viral diseases and results in considerable economic losses in the pork production industry. The disease can be controlled using preventive measures such as improved stock management and vaccination throughout the pig-rearing period. We developed a stochastic model based on Population Dynamics P systems (PDP) models for a standard pig production system to differentiate between the effects of pig farm management regimes and vaccination strategies on the control of Aujeszky’s disease under several different epidemiological scenarios. Our results suggest that after confirming the diagnosis, early vaccination of most of the population (>75%) is critical to decrease the spread of the virus and minimize its impact on pig productivity. The direct economic cost of an outbreak of Aujeszky’s disease can be extremely high on a previously uninfected farm (from 352–792 Euros/sow/year) and highlights the positive benefits of investing in vaccination measures to control infections. We demonstrate the usefulness of computational models as tools in the evaluation of preventive medicine programs aimed at limiting the impact of disease on animal production.

## 1. Introduction

According to the United Nations Food and Agriculture Organization (FAO), food production must increase by 70% to feed the world’s population by the year 2050 [1]. A sustained increase in pig production will be necessary to cope with this challenge and provide enough pork worldwide, by increasing the number of animals in production and/or improving the efficiency of the sector. Increasing the number of animals worldwide is problematic due to environmental restrictions as well as the decrease in pig production in certain countries [2]. Consequently, optimizing the efficiency of pig production is essential in the short run. Production efficiency globally suffers from the multiple effects of infectious and non-infectious diseases, such as mortality losses, reduced feed conversion ratio, increased veterinary costs, and the lost or lowered value of infected carcasses [3]. It is therefore necessary to evaluate the economic consequences of disease on animal performance in order to select the best husbandry practices based on cost/benefit analyses [4].

Many pig diseases cause considerable economic losses for the swine industry [5]. Most are disease complexes, such as the viral/bacterial respiratory disease complexes occurring during the rearing period [6]. Preventive medicine programs can control viruses and/or bacteria in various ways, such as gilt acclimation protocols, vaccination programs for gilts and sows, various management protocols to control pathogens in suckling pigs, and vaccination protocols in growing animals [4,7,8]. This last measure requires case-by-case evaluation due to possible interactions between the maternally derived immunity and the active immunization of their offspring [9]. Under practical conditions, disease control measures are usually applied in parallel (e.g., management and vaccination), which makes it extremely difficult to disentangle the exact roles and efficiency of each particular measure.

Animal health can mainly be improved by management procedures and vaccination programs focused on limiting the transmission of infectious agents within the farm population. For example, basic management, such as reducing the mixing of animals during the rearing cycle, can decrease the spread of porcine reproductive and respiratory syndrome virus (PRRSV) and, consequently, reduce its impact on pig productivity [10]. However, it is difficult to obtain scientific information on the effectiveness and economic benefits of such measures under field conditions. Similarly, although vaccines are initially evaluated in vaccination-challenge experiments to determine the protection conferred after infection and the amount of pathogen excreted, these experiments do not shed light on how transmission occurs, as they focus on the individual pig and not on the population as a whole [11]. Vaccination reduces the susceptibility of an individual to infection and reduces its subsequent infectivity after infection, and this reduced susceptibility and infectivity, called herd immunity, is certainly of benefit to the population. The chances of an individual becoming infected decrease as the density of vaccinated animals in a population increases [12]. In pig medicine, the prevalence of Aujeszky’s disease has been reduced in many countries by vaccination strategies combined with the elimination of infected animals, and the available vaccines are well-known and highly efficacious [11,13].

Mathematical models can help us to understand the factors influencing the epidemiology of infectious diseases and to design more efficient control strategies [14]. They allow the construction of ’what if’ scenarios to predict the effects of various interventions and the likely disease dynamics and transmission outcomes [15,16,17]. To date, this type of modeling has usually been carried out using stochastic models for other pig diseases [18,19,20]. Previously, we developed a Population Dynamics P system (PDP) model (inspired by the biology of cell function) to model the effect of management procedures on the productivity of Porcine Reproductive and Respiratory Syndrome Virus (PRRSV)-infected farms [10]. Briefly, cells can perform multiple processes simultaneously in a synchronized fashion, making them a suitable example for the modelling of complex problems. New emerging generations of computational models such as PDP models are useful tools for the study of complex problems, handling a huge number of interactions in a more efficient fashion [21]. There have been many publications studying the effect of vaccination on the epidemiology of Aujeszky’s disease at the farm and regional levels [22,23]. Unfortunately, there have been no studies of factors affecting the epidemiology of infectious diseases, such as management procedures in combination with vaccination as tools for the control of this disease. The main goal of this paper is to develop a stochastic model, based on our PDP model for PRRSV, to mimic the intra-herd dynamics of a standard pig production system in order to disentangle the effects of pig farm management measures and vaccination strategies in the control of Aujeszky’s disease, under several different epidemiological scenarios.

## 2. Materials and Methods

### 2.1. Ethical Statement

No animals were used in this research, due to its entirely theoretical approach.

### 2.2. Farm Structure and Management Procedures

A previously published stochastic PDP model for a swine breeding herd describes the spatial organization of a typical herd and takes all of the production stages (sows, piglets and pigs) into account [10]. Here we only model the nursery and fattening phases of the process. The nursery phase contains pigs from 3–9 weeks of age and is followed by the final fattening phase, when the pigs are from 9–24 weeks of age. The animals are moved between phases and sub-population groups on a weekly basis and are assumed to mingle homogenously within their respective production units. In each of the production phases (nursery and fattening), a series of management decisions is taken based on the development and physiological condition of the animals. The nursery phase begins when piglets are separated from their mothers and allocated to pens according to different management regimes: (i) grouping piglets according to the identity of their mothers, and (ii) allocating the piglets randomly by pen. In both cases, piglets are separated according to sex. During the fattening phase, animals are moved to fattening pens located in other buildings. We examined the extremes of management of this movement: (i) orderly movements whereby the fattening pens were filled according to the identity of the piglets’ mothers and their distribution in the nursery (labelled ‘MP’ or ‘mother’ in the figures), or (ii) random movements whereby nursery and fattening pens were filled with piglets at random (RR or ‘random’ in the figures).

### 2.3. Population Dynamics P System (PDP) Model

The PDP model used [10] allows the effects of management and vaccination practices (percentage of animals vaccinated, timing of vaccination and vaccine efficiency) to be studied under different levels of infection on an integrated farm (Table S1). The model was run 30 times for a farm with 1000 sows, taking into account the epidemiological parameters associated with Aujeszky’s disease obtained from the literature (Table 1). The basic reproductive ratio was set at 10, being towards the high end of the range described in the literature, in order to develop a worst-case scenario [24]. The annual piglet production was close to the average value for a sow farm in Spain (27.5 piglets weaned/sow/year) [25].

### 2.4. Epidemiological Scenarios for Aujeszky Virus

We used a PDP model to assess the effects of the Aujeszky virus on the economic and animal production for a previously Aujeszky-free farm (and therefore with no maternally derived immunity in piglets) that had become infected during the nursery phase due to a breach in biosecurity. We simulated the effectiveness of vaccination in controlling the disease over a production cycle under four scenarios: (i) a low infection rate (5% of the population), (ii) a 10% infection rate, (iii) a 15% infection rate and (iv) a high infection rate (20% of the population). The efficacy of the vaccine was 90% according to the available literature [24]. The vaccine can be administered at 1, 10, 20, 30 or 40 days after the beginning of the nursery period (assumed to be at the time that the infection was discovered, for the sake of simplicity) and to either 25%, 50%, 75% or 100% of the population. Combining four levels of disease prevalence, two types of management during the nursery and fattening phases (MP and RR), five times at which the vaccine can be administered (1, 10, 20, 30 and 40 days) and the percentage of the population vaccinated (25%, 50%, 75%, and 100%) gives 160 possible combinations of management and vaccination strategies. The model was run 30 times, commencing at the beginning of the nursery period in all of the simulations.

### 2.5. Economic Impact

To assess the economic impact, we calculated the direct cost of lost pigs, taking into account the average number of pigs produced by a pig farm in Spain (27.5 piglets/sow/year), the market average weight of a pig (100 kg) and the average price/kg of pork during 2019 in Spain (€1.34).

### 2.6. Statistical Analyses

All statistical analyses were performed using the R Core package (R Core team (2018), R Foundation for Statistical Computing,Vienna, Austria). The PDP model used to simulate the population’s dynamics under different infection, management and vaccination regimes has been published previously [10]. Two types of statistical analyses were performed on the model results. After an exploratory analysis, the results were quantified objectively using generalized linear models (GLM), with a Poisson family and logarithm as a function link. Analyses were considered to show statistically significant differences at *p*-values <0.05.

The independent variables in the model were the proportion of Aujeszky-infected piglets at the beginning of the nursery period (or at the time of infection), the lag time between infection and vaccination of piglets, the percentage of the population vaccinated and the management procedure applied during the nursery and fattening phases (MP or RR). The dependent variables were the percentage of viable animals, the percentage of sick animals and the percentage total weight loss of animals (each value both at the end of the nursery and fattening period).

**Figure 1:**
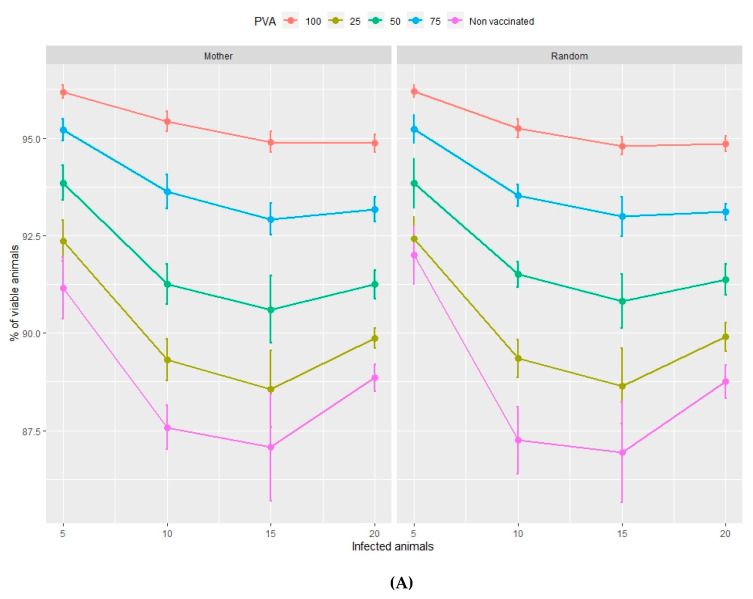

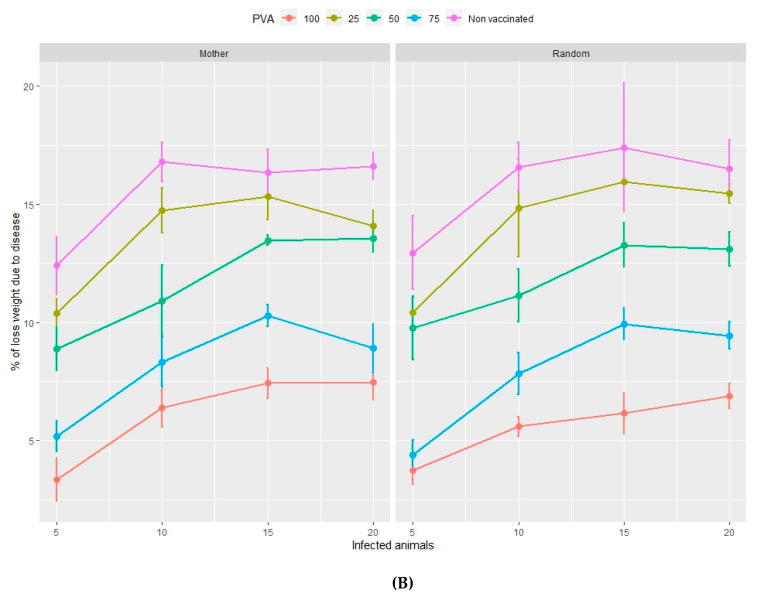
Percentage of viable animals (**A**) and percentage of total weight lost (**B**) at the end of the nursery period in relation to the type of management (mother or random), the percentage of vaccinated animals (PVA—ranging from 0%–100% of the population) and the percentage of infected animals at the time of vaccination. Orderly movements of piglets consist of allocating them to nursery pens according to the identity of their mothers and then filling the fattening pens according to the same nursery distribution (Mother) c.f. random movements whereby piglets are allocated to both nursery and fattening pens at random (Random).

**Figure 2:**
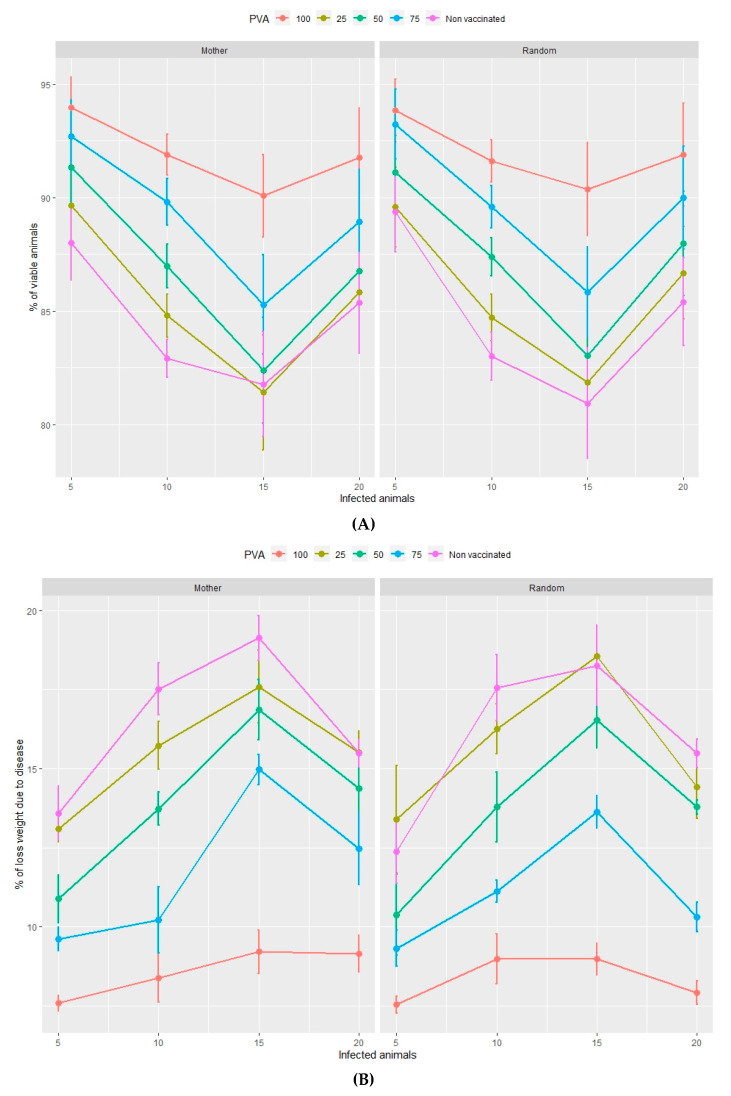
Percentage of viable animals (**A**) and percentage of total weight loss (**B**) at the end of the fattening period in relation to the type of management (mother or random), the percentage of vaccinated animals (PVA—ranging from 0%–100% of the population) and the percentage of infected animals at the time of vaccination. Orderly movements of piglets consists of allocating them to nursery pens according to the identity of their mothers and then filling the fattening pens according to the same nursery distribution (Mother) c.f. random movements whereby piglets are allocated to both nursery and fattening pens at random (Random).

**Figure 3:**
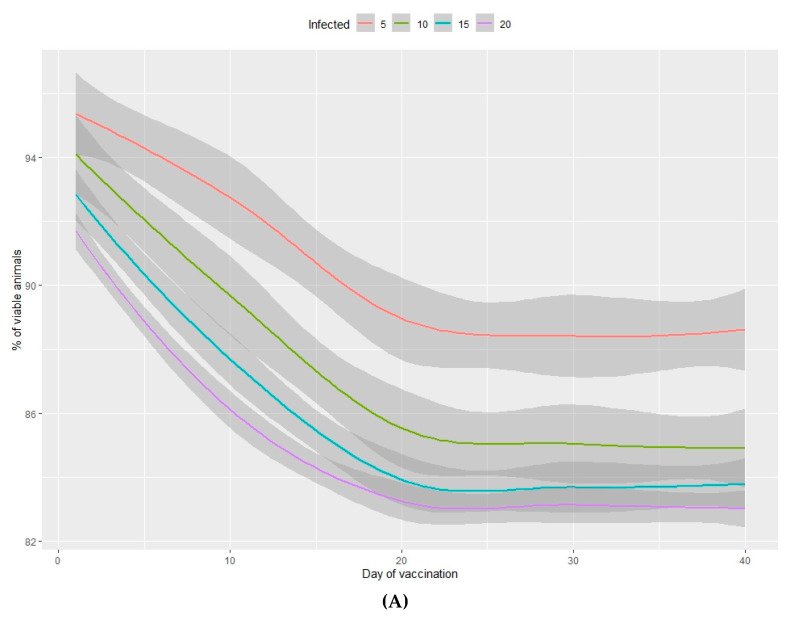

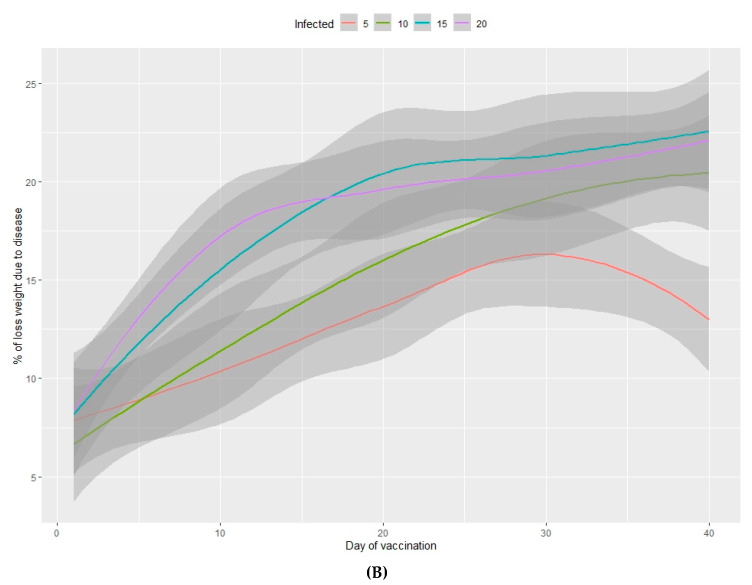
Percentage of viable animals (**A**) and percentage of total weight lost (**B**) at the end of the nursery period in relation to the percentage of infected animals (from 5%–20%) and the number of days between infection and vaccination of 100% of the population.

**Figure 4:**
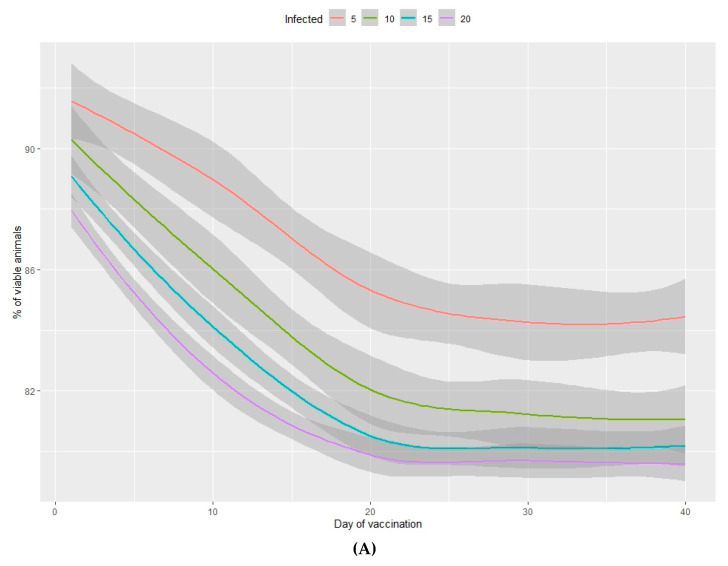

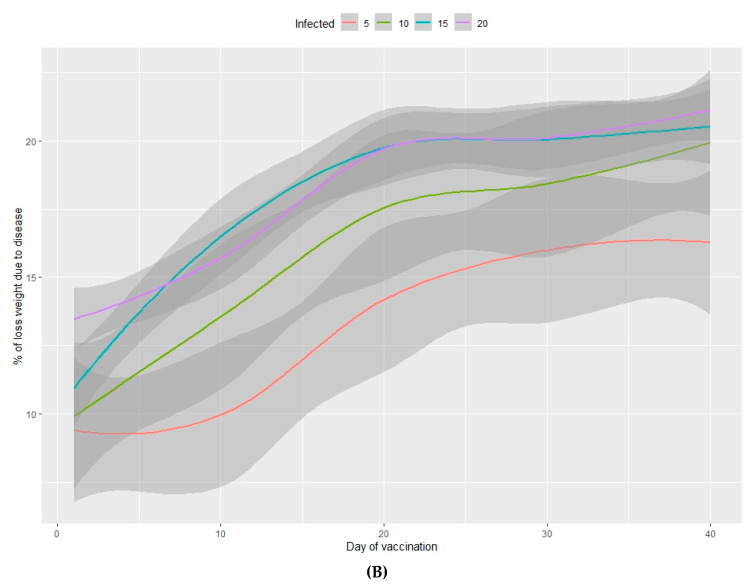
Percentage of viable animals (**A**) and percentage of total weight loss (**B**) at the end of the fattening period in relation to the percentage of infected animals (from 5%–20%) and the number of days between infection and vaccination of 100% of the population.

The output values of the dependent variables produced by each of the simulations are shown in Figure 1, Figure 2, Figure 3 and Figure 4 and in the Supplementary Material Figures S1 and S2.

## 3. Results

### 3.1. Descriptive Results

The percentage of viable animals and the loss of total weight, at the end of both the nursery (Figure 1A,B) and fattening (Figure 2A,B) phases, significantly increase and decrease, respectively, as the percentage of vaccinated animals increases (from 0%–100%). This pattern is repeated with rising percentages of infected animals at the beginning of the vaccination process in the range of 5%–10% infection. However, both variables show no significant trends if the percentage of infected animals is higher than 10%, in either the nursery or the fattening phase. According to Figure 1 and Figure 2, the effect of the management regime is apparently negligible compared to the other factors studied.

The percentage of viable animals produced and the loss of total weight at the end of the production period are shown in Figure 3A,B (nursery phase) and Figure 4A,B (fattening phase) in relation to the percentage of infected animals at the beginning of the nursery period (time of infection) and the lag time before vaccination of 100% of the population. In all cases, the higher the percentage of infected animals at the beginning of the nursery phase, the worse the production performance during the rearing period. Moreover, the earlier the vaccine is administered in the population, the better the production performance obtained during the nursery and fattening phases. Even a one-day delay in administering the vaccine can result in a loss of 0.2% of animals daily and a consequent loss of total weight at the end of the nursery phase. A 10-day delay in administering the vaccine implies a 5% decrease in animals remaining at the end of the fattening phase, when the starting point is 5% of sick animals. When 20% of animals are sick, the losses can reach 10% and can vary from 2%–7%, depending on the animals infected. When the vaccine is administered later than 20 days after diagnosing the disease, there is no observed significant improvement in any of the measured variables.

The percentage of sick animals at the end of the nursery phase increases in parallel with the percentage of infected animals at the beginning of the nursery phase. The contrary is observed regarding the percentage of vaccinated animals in the population (Figure S1). In relation to the fattening phase (Figure S2), the percentage of sick animals at the end of this phase decreases inversely to the percentage of infected animals at the beginning of the nursery phase. A reasonable explanation could be based on the quick dissemination of the disease during the nursery phase at high levels of infection and low levels of vaccination, whereby most of the animals contract the disease during the nursery phase.

### 3.2. GLM Model

Table 2 and Table 3 show the results of the GLM models. The effect of management is not statistically significant (i.e., *p* > 0.05). In the models studied, there are two important factors in the spread of Aujeszky’s disease: the percentage of animals vaccinated, and the period elapsed between infection and vaccination.

#### 3.2.1. Viability of Animals

In the nursery phase, the percentage of viable animals is mainly affected by the percentage of animals that are vaccinated at the time at which the disease is detected. The difference between vaccinating 75% or 100% of animals is not statistically significant (Table 2). The number of viable animals at the end of the nursery phase is between 95% and 96% of those entering this phase of production (Figure 1A, Table S2). Animals that reach the end of the fattening phase are affected by both the percentage of animals vaccinated and the percentage of sick animals (Table 2, Figure 2A). The differences observed between vaccinating 75% or 100% of the animals are still not significant during this phase. Where 100% of the animals are vaccinated, the percentage of viable animals at the end of the fattening phase is between 92% and 94%, depending on the percentage of sick animals at the beginning of the nursery phase (Figure 2B, Table S2). For each day that vaccination is delayed, an average loss of 0.2% of animals is estimated, both in the nursery and fattening phases (Figure 3A and Figure 4A).

#### 3.2.2. Loss of Total Weight Due to Disease

Total weight loss at the end of both the nursery and fattening phases is significantly affected by the percentage of infected animals and the percentage of vaccinated animals (Table 2, Figure 1B). The minimum average total weight loss in the nursery phase is 3.5%, in the case where 5% of the animals are sick and where 100% of the animals are vaccinated (Table S3). If 75% of the animals are vaccinated, the loss increases by more than 1%.

In the fattening phase, these total weight losses increase substantially, and the minimum loss is estimated at an average of 7.5%. A farm with 1000 sows is estimated to produce about 27,500 animals on average, each with an average weight of 100 kg at the end of the fattening cycle. Based on these assumptions, weight losses of 7.5% (Figure 2B, Table S2) reduce production by 206,250 kg of meat with a direct economic loss of €280,500/year. Where 100% of the animals are vaccinated, the annual economic loss depends on the number of days delay in administering the vaccine and the percentage of infected animals and varies between €352,173 and €790,447 (Table 4).

The fattening phase is the last phase of the pork production process, so these total weight losses translate directly into decreased income and indirectly into decreased business profits. The average loss for each day that vaccination is delayed is greater than 0.2% (Table S3), the equivalent of 5500 kg of meat daily with an economic loss of €7480/day, assuming that all sub-populations are infected.

## 4. Discussion

A sustained increase in pig production is necessary to cope with the challenge of providing enough pork worldwide in the future. Improving production efficiency will be the goal for pig production in the short run due to legal restrictions on increasing the number of animals in many pig-producing countries. Mathematical models can help us to understand the factors influencing the epidemiology of infectious diseases and to design more efficient control strategies. The Susceptible–Infectious–Recovered (SIR) model was initially used for modelling pseudorabies epidemiology at farm and regional levels [13]. The SIR model is a stochastic and equation-based model where animals are sorted into population groups where any animal has the same chance of catching the pathogen from an infected animal due to the homogeneous mixing in the population. This situation is not usual in pig farms because animals are located in pens. In our approach, we have used a stochastic and more advanced model (PDP) [10,21], which works with individuals (agent-based model) in which each pig moves around and acts according to their own specific rules and is grouped into smaller groups (by pen, for example) in order to better mimic the intra-herd dynamics of a standard pig production system [10]. Accordingly, our results suggest that PDPs can provide an important tool for forecasting animal production trends and improving management decisions to reduce economic losses.

Infectious diseases are one of the main factors damaging pig production efficiency and have multiple effects on the global output of pork at the farm level [3]. Preventive medicine programs are targeted at the control of viruses and/or bacteria using various tools such as gilt acclimation protocols, vaccination programs for gilts and sows, various management protocols to control pathogens in suckling pigs and, finally, vaccination protocols for growing animals [4,7,8]. In practice, several measures are usually applied in parallel (e.g., management and vaccination measures), making it extremely difficult to disentangle the exact role of each particular measure in controlling diseases. This problem is almost impossible to resolve experimentally due to the extreme complexity of the experimental design required, hence the importance of the results obtained using our PDP model—results that allow the analysis of theoretical scenarios to evaluate the impact of different measures to control infectious diseases under practical conditions. According to the results of our simulation model, a basic management measure, such as reducing the mixing of animals during the rearing cycle, seems to have no significant impact on the spread of the Aujeszky virus and, consequently, does not reduce its impact on pig productivity. This result is contrary to recently published information for PRRSV [10]. A reasonable explanation for this difference could be the different basic reproduction ratio (R0) observed between PRRSV and the Aujeszky virus. The R0 of Aujeszky’s disease (10) is higher than that of PRRSV (3.5) [10,24]. For this reason, management procedures could be more relevant in infectious disease control for less contagious diseases. Our conclusion is based on pseudorabies transmission inside each farm, and it cannot be extrapolated to pseudorabies transmission between herds where other factors, such as the herd size, are relevant [13]. Anyway, this conclusion needs to be checked against other pig diseases using modelling approaches.

Vaccines are often used to control pig diseases and are initially evaluated in vaccination-challenge experiments to determine the clinical protection conferred after infection and the amount of pathogen excreted, but these experiments do not shed light on how transmission occurs, as they focus on the individual pig and not on the whole population [3]. In many countries, a combination of vaccination strategies and the elimination of infected animals has managed to decrease the prevalence of Aujeszky’s disease [22,23]. There is a good deal of published evidence regarding the beneficial effect of vaccination on the epidemiology of Aujeszky’s disease at farm and regional level because of its importance in the control and/or eradication of this viral disease, and the vaccine is therefore well-known for its high level of efficacy. Our results support the positive effect of vaccination in controlling Aujeszky’s disease infection but show that the extent of the benefits depends on the percentage of the population vaccinated and the time lag between infection and vaccination. Our results clearly highlight the importance of early vaccination as soon as the infection is confirmed. In practice, vaccination has been a common clinical practice for swine veterinarians when an Aujeszky outbreak is suspected, and our results clearly support this intervention and agree with other published information for other animal diseases [26,27,28,29].

With respect to the economic impact, we provide a simple estimate of the direct costs due to lost animal weight and its direct consequences for farm revenue. Our approach does not intend to provide an exhaustive cost analysis of Aujeszky’s disease outbreaks but only to make rough estimates of the economic consequences. According to our calculations, the cost of the disease could be extremely high for an outbreak on a previously unaffected farm (from €352 to €792/sow/year), as shown in our simulation model, assuming that every phase of production is affected by the disease. These results agree with information previously published by other authors. Such heavy losses are the reason that pig-producing countries have all carried out eradication programs to get rid of this disease [30,31,32,33].

## 5. Conclusions

Our model was able to disentangle the impacts of different measures to control an outbreak of Aujeszky’s disease. Thus, our results suggest that after confirming the diagnosis, early vaccination of most of the population (>75%) is critical to decrease the spread of the virus and minimize its impact on pig productivity. However, the effect of management is negligible for the control of this virus under our conditions. The development of this PDP model could be especially relevant for new pig diseases, where information is limited, and such models could be an important tool for policy decision makers.

## Figures and Tables

**Table 1:** Epidemiological parameters used in a Population Dynamics P system model of the effects of different management and vaccination approaches to control Aujeszky disease on a 1000-sow farm.

**Non-Vaccinated**			
Parameter	Lactation period	Nursery	Fattening
Basic reproductive ratio (Ro)	NA	10	10
Incubation period (days)	NA	4	4
Infectious period (days)	NA	15	15
Lethality	NA	15	10
Reduction of weight at the end of the period (gr)		1000	4000
**Vaccinated**			
Parameter	Lactation period	Nursery	Fattening
Basic reproductive ratio (Ro)	NA	0.5	0.5
Incubation period (days)	NA	4	4
Infectious period (days)	NA	10	10
Lethality	NA	6	3
Reduction of weight at the end of the period (gr)		500	2000

NA: Non-applicable.

**Table 2:** Generalized Linear Model (GLM) results. The effects of different percentages of infected and vaccinated animals and management regimes on the percentages of viable and sick animals, and of animals showing total weight loss, during the nursery and fattening phases of production. Two management regimes are compared: orderly movements, in which fattening pens are filled with piglets housed together in the nursery unit according to the identity of their mother (mother management); and random movements, where piglets are assigned to the nursery and fattening pens at random (random management).

GLM Models	Estimate	*p*-Value	Estimate	*p*-Value
% of viable animals	Nursery		Fattening	
(Intercept)	4.578	<0.001	4.559	<0.001
Infected 10	−0.026	0.0892	−0.045	0.004
Infected 15	−0.033	0.0350	−0.080	<0.001
Infected 20	−0.024	0.1178	−0.036	0.022
25% of vaccinated animals	−0.057	0.0011	−0.072	<0.001
50% of vaccinated animals	−0.037	0.0300	−0.054	0.002
75% of vaccinated animals	−0.017	0.327	−0.028	0.115
Non vaccinated	−0.072	<0.001	−0.083	<0.001
Random management	0.0004	0.971	0.003	0.770
Deviance of model	89.99%		69.79%	
% of sick animals	Nursery		Fattening	
(Intercept)	2.303	<0.001	0.107	0.412
Infected 10	0.364	<0.001	0.063	0.073
Infected 15	0.485	<0.001	−1.650	<0.001
Infected 20	0.5601	<0.001	−0.547	<0.001
25% of vaccinated animals	1.774	<0.001	3.051	<0.001
50% of vaccinated animals	1.576	<0.001	3.091	<0.001
75% of vaccinated animals	0.998	<0.001	2.531	<0.001
Non vaccinated	1.829	<0.001	2.807	<0.001
Random management	0.002	0.905	0.143	<0.001
Deviance of model	96.06%		75.45%	
% of loss weight due to disease	Nursery		Fattening	
(Intercept)	1.464	<0.001	1.9545	<0.001
Infected 10	0.328	<0.001	0.2126	<0.001
Infected 15	0.433	<0.001	0.3552	<0.001
Infected 20	0.404	<0.001	0.1792	<0.001
25% of vaccinated animals	0.862	<0.001	0.6088	<0.001
50% of vaccinated animals	0.695	<0.001	0.4875	<0.001
75% of vaccinated animals	0.314	<0.001	0.3019	<0.001
Non vaccinated	0.984	<0.001	0.6465	<0.001
Random management	0.002	0.939	−0.0249	0.393
Deviance of model	88.38%		88.45%	

**Table 3:** Generalized Linear Model results. The effects of the percentage of infected animals, the day of vaccination, and management regime on the percentages of viable and sick animals, and animals showing total weight loss during the nursery and fattening phase when 100% of the animals are vaccinated. Two management regimes are compared: orderly movements, in which fattening pens are filled with piglets housed together in the nursery unit according to the identity of their mother (mother management); and random movements, where piglets are assigned to the nursery and fattening pens at random (random management).

GLM Models	Estimate	*p*-Value	Estimate	*p*-Value
% of viable animals	Nursery		Fattening	
(Intercept)	4.560	<0.001	4.518	<0.001
Infected 10	−0.033	0.483	−0.033	0.501
Infected 15	−0.050	0.292	−0.048	0.319
Infected 20	−0.061	0.200	−0.059	0.227
Day of vaccination	−0.002	0.049	−0.003	0.043
Management	−0.006	0.854	−0.006	0.863
Deviance of model	83.37%		84.63%	
% of sick animals	Nursery		Fattening	
(Intercept)	3.285	<0.001	0.654	0.014
Infected 10	0.319	<0.001	−0.446	0.045
Infected 15	0.438	<0.001	−0.989	<0.001
Infected 20	0.508	<0.001	−1.503	<0.001
Day of vaccination	0.026	<0.001	0.044	<0.001
Management	0.047	0.214	−0.140	0.454
Deviance of model	74.95%		79.12%	
% of loss weight due to disease	Nursery		Fattening	
(Intercept)	2.072	<0.001	2.272	<0.001
Infected 10	0.186	0.129	0.193	0.102
Infected 15	0.364	0.002	0.273	0.019
Infected 20	0.362	0.002	0.322	0.005
Day of vaccination	0.019	<0.001	0.013	<0.001
Management	0.017	0.832	0.031	0.694
Deviance of model	74.34%		79.62%	

**Table 4:** Annual direct economic losses (Euros) for a 1000-sow farm (mean ± SD) in relation to the number of days between infection and vaccination and the percentage of sick animals at the beginning of the nursery phase. It is assumed that the disease affects every production batch.

Day of Vaccine Application	Percentage of Sick Animals			
	5%	10%	15%	20%
1	352,173 ± 29,500	370,655 ± 32,632	409,303 ± 2301	504,044 ± 26,193
10	372,651 ± 7247	506,993 ± 10,497	616,843 ± 17049	587,593 ± 28,184
20	530,288 ± 60,536	655,955 ± 38,887	738,466 ± 16,356	736,997 ± 12,592
30	598,236 ± 33,832	689,390 ± 65,294	749,734 ± 37,314	752,063 ± 27,830
40	608,803 ± 85,860	745,715 ± 79,878	768,040 ± 40137	790,447 ± 4899

## Supplementary Materials

The following are available online at https://www.mdpi.com/2076-2615/10/5/909/s1: Figure S1: Nursery sick (A) and fattening sick animals (B), depending on the type of management, the percentage of infected animals at vaccine application and the percentage of vaccinated animals; Figure S2: Nursery sick (A) and fattening sick animals (B), depending on the day of vaccination and the percentage of infected animals when the vaccine is applied to 100% of the population; Table S1: Production parameters used to run the model in a 1000-sow farm; Table S2: Average values of viable, sick animals and the consequent weight loss at the end of the nursery and fattening period depending on the percentage of infected and vaccinated animals; Table S3: Average values of viable, sick animals and the consequent weight loss at the end of the nursery and fattening period depending on the percentage of infected and the day of vaccination of the whole population (100% of the animals) versus the infection day.
