# Supplementary material for: Vaccination Is a Suitable Tool in the Control of Aujeszky’s Disease Outbreaks in Pigs Using a Population Dynamics P Systems Model
Source: Animals (Basel). 2020 May 24;10(5):909. doi: 10.3390/ani10050909 (PMC7278389; doi:10.3390/ani10050909)

**SUPPLEMENTARY MATERIAL**

**Tables**

**Table S1**. Production parameters used to run the model in a 1000 sow farm.

| | | |
|---|---|---|
| Mothers | Batches | 20 |
| | Number sows per batch | 50 |
| | Fertility to delivery | 0.85 |
| | Fertility at gestation control | 0.9 |
| | Abortion probability | 0.02 |
| | Average number of live offspring | 12.55 |
| | Average number of stillborn piglets | 1.5 |
| | Sow mortality | 0.06 |
| | Maximum number of deliveries by mother | 8 |
| | Maximum number of failures by mother | 3 |
| | Minimum number of failures by mother to remove | 2 |
| | Maxim parity to be removed | 5 |
| | Annual sow replacement rate | 0.49 |
| Lactation period Piglet data | Mortality week 1 | 0.06 |
| | Mortality week 2 | 0.04 |
| | Mortality week 3 | 0.02 |
| Transition period | Number of pen | 22 |
| | Mortality | 0.03 |
| Fattening period | Number of pen | 44 |
| | Mortality | 0.04 |

**Tabla S2.** Average values of viable, sick animals and the consequent weight loss at the end of the nursery and fattening period depending on the percentage of infected and vaccinated animals.

| Nursery | % Infected animals | Vaccination day | | | | | Fattening | % Infected animals | Vaccination day | | | | |
|---|---|---|---|---|---|---|---|---|---|---|---|---|---|
| | | 0 | 25 | 50 | 75 | 100 | | | 0 | 25 | 50 | 75 | 100 |
| % of viable animals | 5 | 91.57 | 92.39 | 93.84 | 95.22 | 96.19 | % of viable animals | 5 | 88.71 | 89.62 | 91.22 | 92.97 | 93.91 |
| | 10 | 87.42 | 89.33 | 91.38 | 93.58 | 95.33 | | 10 | 82.96 | 84.76 | 87.19 | 89.71 | 91.75 |
| | 15 | 87 | 88.60 | 90.71 | 92.95 | 94.85 | | 15 | 81.36 | 81.63 | 82.71 | 85.56 | 90.23 |
| | 20 | 88.80 | 89.89 | 91.31 | 93.14 | 94.86 | | 20 | 85.39 | 86.27 | 87.37 | 89.48 | 91.84 |
| % of sick animals | 5 | 71.40 | 63.96 | 44.75 | 20.66 | 6.09 | % of sick animals | 5 | 26.62 | 28.96 | 23.32 | 8.47 | 0.44 |
| | 10 | 93.55 | 86.82 | 69.32 | 35.98 | 12.08 | | 10 | 21.73 | 28.87 | 29.23 | 12.91 | 0.76 |
| | 15 | 98.22 | 94.71 | 80.21 | 45.75 | 17.10 | | 15 | 3.19 | 1.07 | 3.03 | 8.40 | 1.19 |
| | 20 | 99.37 | 97.47 | 87.09 | 55.49 | 22.94 | | 20 | 4.72 | 12.91 | 19.21 | 12.94 | 1.02 |
| % of loos weight due to disease | 5 | 12.68 | 10.40 | 9.33 | 4.79 | 3.54 | % of loos weight due to disease | 5 | 12.98 | 13.25 | 10.63 | 9.46 | 7.56 |
| | 10 | 16.69 | 14.79 | 11.02 | 8.08 | 5.99 | | 10 | 17.53 | 15.99 | 13.76 | 10.67 | 8.69 |
| | 15 | 16.88 | 15.65 | 13.37 | 10.11 | 6.79 | | 15 | 18.69 | 18.07 | 16.71 | 14.30 | 9.10 |
| | 20 | 16.57 | 14.76 | 13.34 | 9.18 | 7.16 | | 20 | 15.48 | 14.98 | 14.08 | 11.39 | 8.53 |

**Table S3.** Average values of viable, sick animals and the consequent weight loss at the end of the nursery and fattening period depending on the percentage of infected and the day of vaccination of the whole population (100% of the animals) versus the infection day.

| Nursery | Vaccination day | | | | | | Fattening | Vaccination day | | | | | |
|---|---|---|---|---|---|---|---|---|---|---|---|---|---|
| | % Infected animals | 1 | 10 | 20 | 30 | 40 | | % Infected animals | 1 | 10 | 20 | 30 | 40 |
| % of viable animals | 5 | 95.36 | 92.75 | 88.96 | 88.41 | 88.62 | % of viable animals | 5 | 91.55 | 88.97 | 85.33 | 84.27 | 84.45 |
| | 10 | 94.09 | 89.68 | 85.53 | 85.04 | 84.91 | | 10 | 90.28 | 86.02 | 82.05 | 81.23 | 81.07 |
| | 15 | 92.83 | 87.69 | 83.93 | 83.68 | 83.79 | | 15 | 89.08 | 84.11 | 80.52 | 80.12 | 80.19 |
| | 20 | 91.69 | 86.12 | 83.25 | 83.13 | 83.01 | | 20 | 87.96 | 82.59 | 79.90 | 79.70 | 79.59 |
| % of sick animals | 5 | 11.10 | 29.53 | 55.68 | 71.72 | 78.03 | % of sick animals | 5 | 0.70 | 1.86 | 2.84 | 7.51 | 12.97 |
| | 10 | 19.90 | 50.92 | 80.45 | 91.74 | 95.45 | | 10 | 1.00 | 2.28 | 2.21 | 4.46 | 6.61 |
| | 15 | 28.43 | 65.50 | 91.31 | 97.18 | 98.83 | | 15 | 1.20 | 2.09 | 1.28 | 2.12 | 2.94 |
| | 20 | 36.82 | 77.01 | 96.03 | 99.26 | 99.86 | | 20 | 1.30 | 1.64 | 0.67 | 0.96 | 1.19 |
| % of loos weight due to disease | 5 | 7.88 | 10.35 | 13.66 | 16.31 | 12.99 | % of loos weight due to disease | 5 | 9.42 | 9.96 | 14.18 | 16.00 | 16.28 |
| | 10 | 6.67 | 11.39 | 16.02 | 19.17 | 20.44 | | 10 | 9.91 | 13.56 | 17.54 | 18.43 | 19.94 |
| | 15 | 8.20 | 15.53 | 20.42 | 21.33 | 22.58 | | 15 | 10.94 | 16.49 | 19.75 | 20.05 | 20.54 |
| | 20 | 8.38 | 17.22 | 19.60 | 20.56 | 22.11 | | 20 | 13.48 | 15.71 | 19.71 | 20.11 | 21.13 |

**Figure S1.** Nursery sick (A) and fattening sick animals (B), depending on the type of management, the percentage of infected animals at vaccine application and the percentage of vaccinated animals.

A

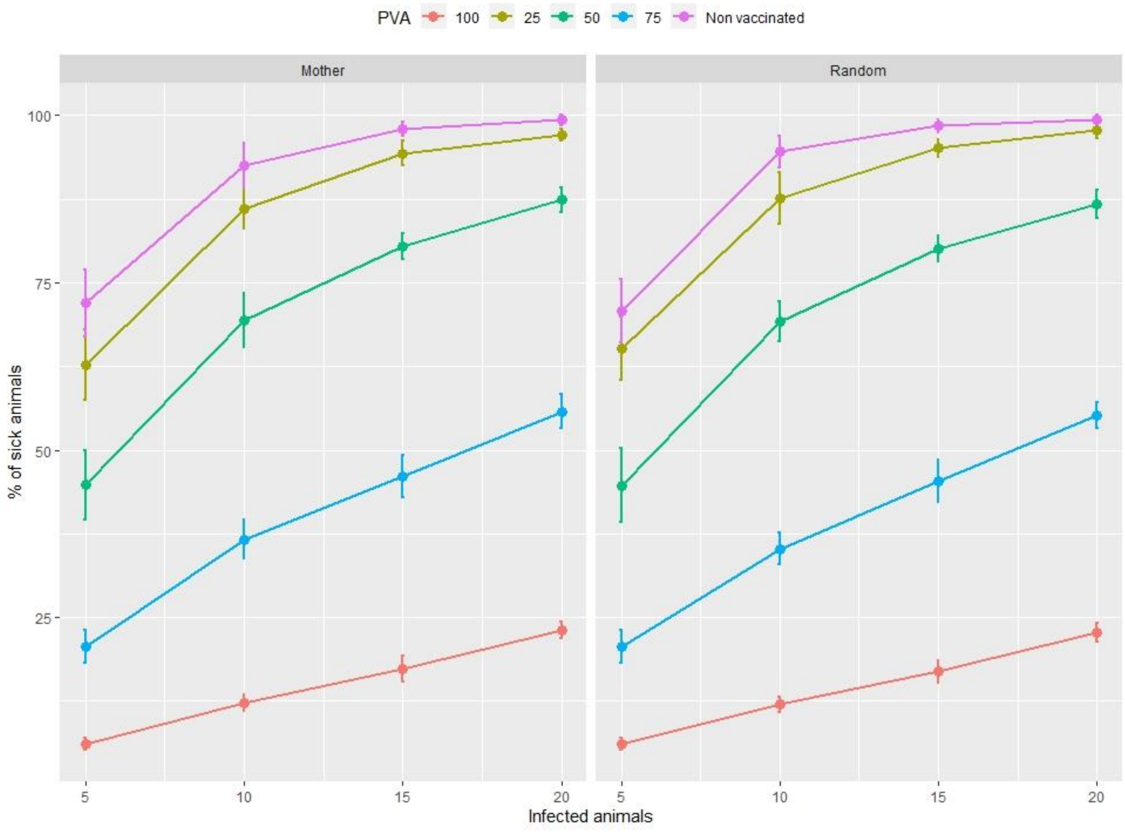


B

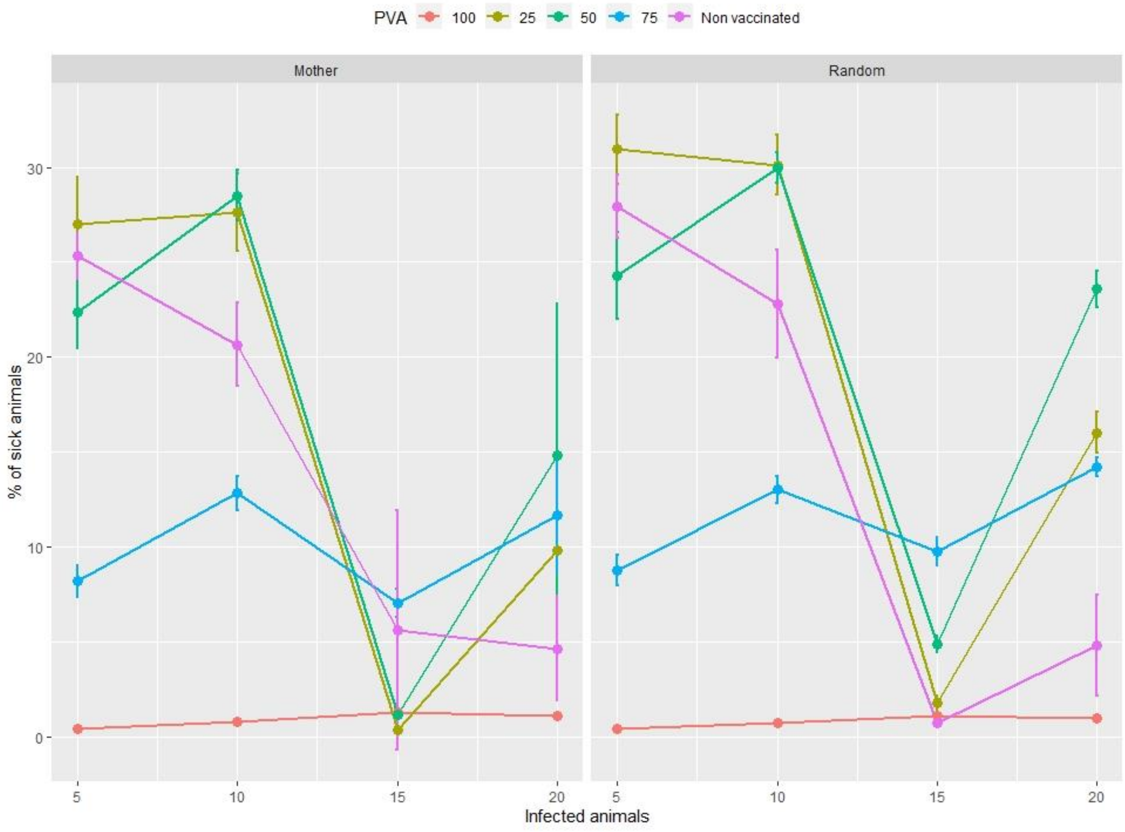

**Figure S2.** Nursery sick (A) and fattening sick animals (B), depending on the day of vaccination and the percentage of infected animals when the vaccine is applied at 100% of the population.

**A**

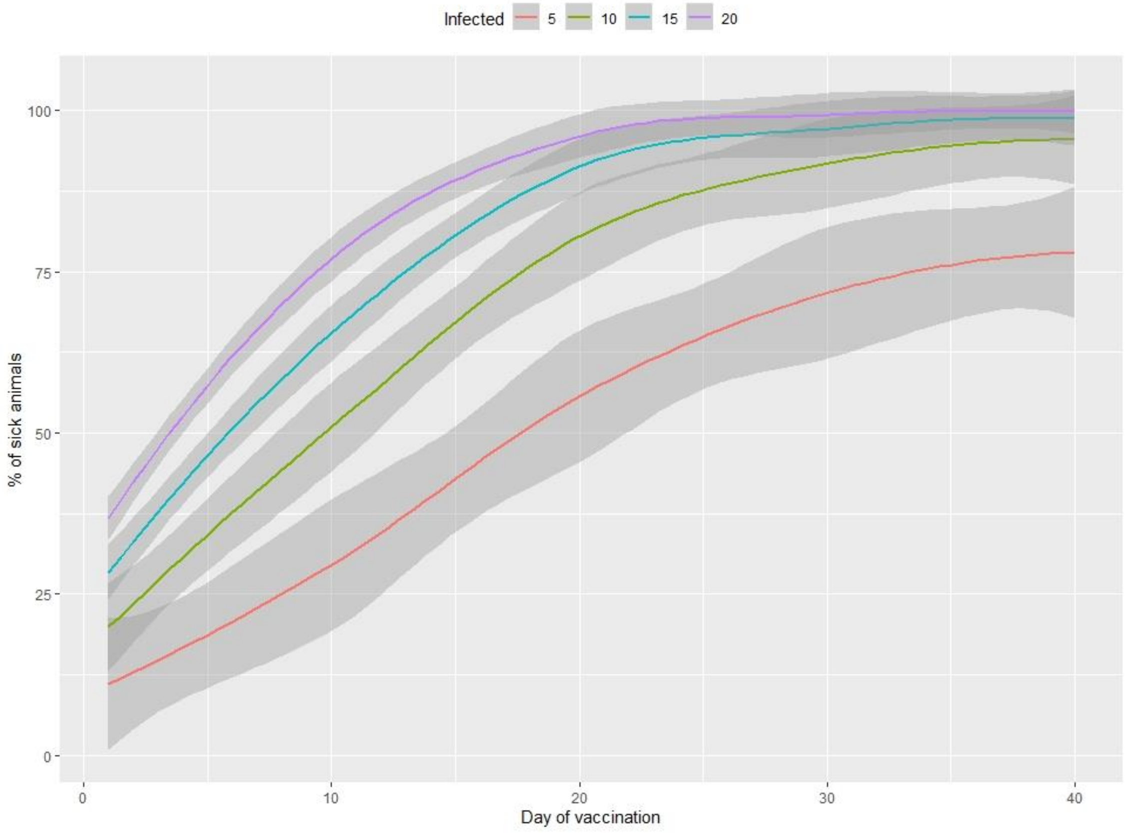


**B**

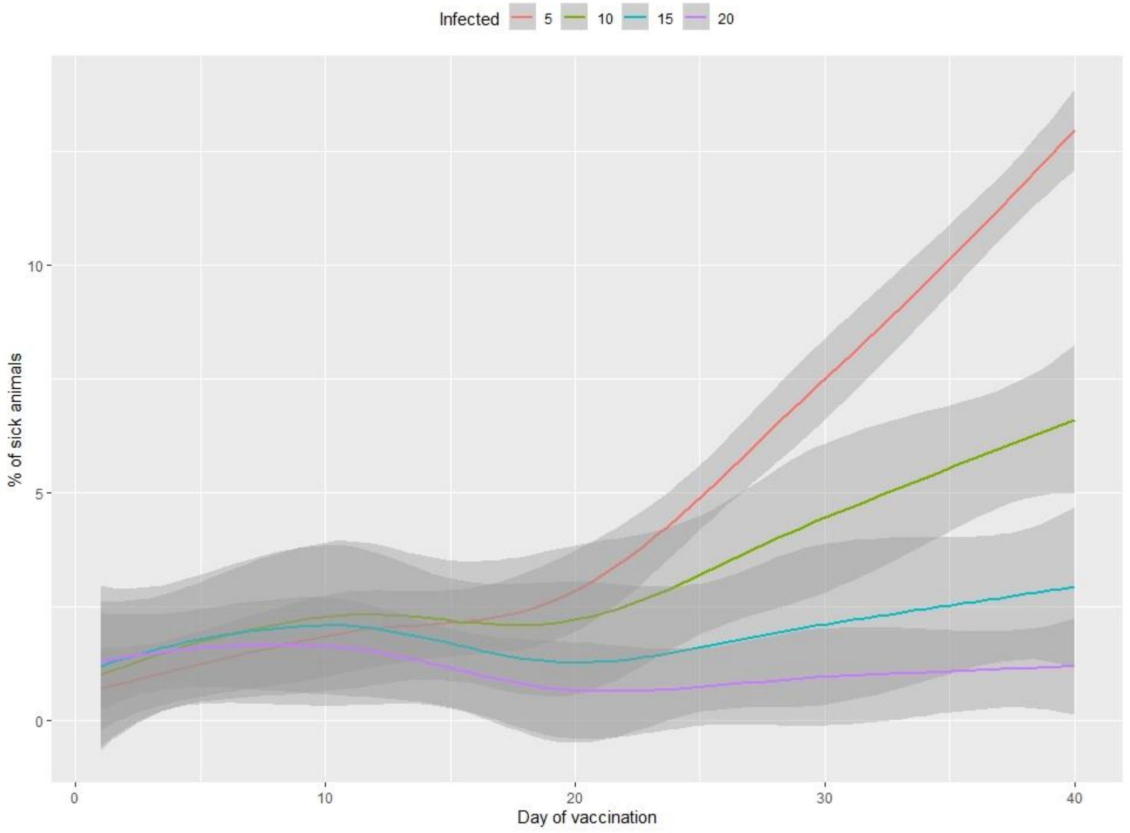

Supplement: Supplementary file 1 [file animals-10-00909-s001.pdf]
